# MBBS admission criteria as predictor of academic performance in a medical college of Pakistan

**DOI:** 10.12669/pjms.39.6.6839

**Published:** 2023

**Authors:** Sundus Iftikhar, Khalid Mahmood Cheema, Iqra Aziz

**Affiliations:** 1 Sundus Iftikhar, M.Phil. Dental Materials, MHPE Department of Medical Education, Shalamar Medical and Dental College, Lahore, Pakistan; 2 Khalid Mahmood Cheema, MS Surgery Department of Medical Education, Shalamar Medical and Dental College, Lahore, Pakistan; 3 Iqra Aziz, MBBS Department of Medical Education, Shalamar Medical and Dental College, Lahore, Pakistan

**Keywords:** Admission criteria, Multiple Mini Interview, cognitive criteria, Non-cognitive criteria, academic performance

## Abstract

**Objective:**

To determine the relationship between scores obtained by students in assessments considered as admission criteria (cognitive and non-cognitive) with their academic performance in medical college.

**Method:**

This correlational study used the data of students who got admission in Shalamar Medical and Dental College (SMDC) in 2015. Spearman correlation and Multiple regression tests were carried out to determine the relationship between admission criteria (Matric, FSC, MDCAT and MMI) and academic performance in medical college (pre-clinical and clinical years).

**Results:**

There was significant positive correlation between scores obtained in MDCAT and pre-clinical years. When combined, MDCAT and MMI scores showed a significant positive correlation with scores obtained by students in clinical years. Scores obtained by students in pre-clinical years strongly correlated with their performance in clinical years. While scores obtained by students in FSC showed negative correlation with clinical year scores, significantly.

**Conclusion:**

It is concluded that the tools used for admission criteria should include both cognitive and non-cognitive elements. MDCAT is a good predictor of academic performance in pre-clinical years however it can only predict performance in clinical years when combined with MMI since MMI assesses the non-cognitive attributes (communication, empathy, ethics etc.) required in those years. FSC should not be given weightage as admission criteria owing to a lot of variability in the exams and scoring of different academic boards of the country

## INTRODUCTION

An admission criterion in a medical college is the first and most important step in selection of competent doctors of the future. For this purpose, medical universities are continuously striving to figure out the best possible criteria that would predict which students would perform well academically in medical school.^1^ Literature shows that commonly, the institutes employ academic tests or pre-entry academic performance as admission criteria.^2^ This makes it necessary to explore the relationship of admission criteria with academic performance of the students. Therefore, several international and local studies have been carried out to explore the relationship of admission criteria with the academic performance of students.

In a study conducted in America it was seen that Medical college entry test when combined with Undergraduate Grade Point Average (UGPA) correlated well with scores of students as they progressed in medical college as well in licensure exams.^3^ A study conducted in Pakistan exploring the relationship of various admission criteria with academic performance in medical college concluded that there was weak correlation between professional exam scores and Matric and FSc scores while MDCAT did not correlate at all.^4^ Another study concluded that that entrance test scores showed positive correlation with scores obtained in MBBS and BDS in initial semesters.^5^

However, a similar study conducted in a nursing institute showed moderately positive correlation between academic performance of students and scores obtained by students at diploma level while weak correlation with entrance test.^6^ Since the current admission criteria in MBBS does not show satisfactory relationship with academic performance, several other attempts have been made to figure out best possible admission criteria. One such criterion includes adding Mathematics in entrance test together with Biology and Chemistry. It showed that adding Mathematics predicted the performance of MBBS better.^7^ Literature suggests adding non-cognitive aspects to admission criteria in addition to cognitive assessment.^8^

One such non-cognitive assessment includes Multiple Mini Interview (MMI). MMI is a type of structured interviews in which a candidate goes through 6-10 stations for interview. Each station of interview is based on a single theme or construct.^9^ Interestingly, Multiple Mini Interview (MMI) has shown to predict academic performance in performance based subjects as well as students’ overall performance (determined via GPA) in a medical school.^10^,^11^ Keeping this in view, Shalamar Medical and Dental College (SMDC) assigned 4% weightage to Multiple Mini Interviews (MMI) scores in 2015 admission criteria in order to assess non-cognitive attributes of applicants. It was believed that since other three criteria all assessed cognition of applicants, MMI would assess the non-cognitive attributes.^12^

**Fig.1:**
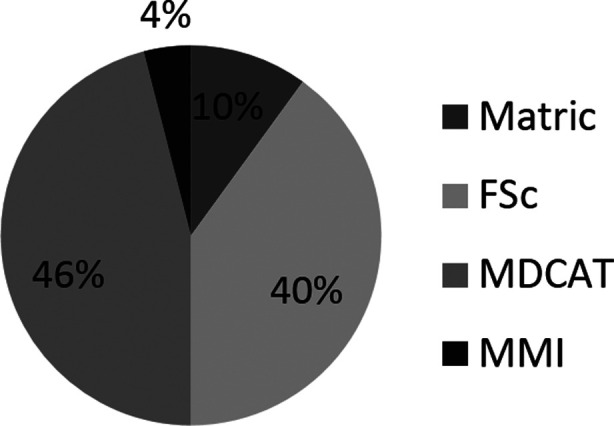
Percentages assigned to various admission criteria in 2015.

In Pakistan, in the year 2015 the merit for medical college was based on aggregate of scores obtained by students in Matriculation (Matric) or Secondary school certificate (SSC), FSc or Higher secondary school certificate (HSSC) (or equivalent scores obtained in O and A level exams), Medical and dental colleges admission test (MDCAT) and individual college’s admission criteria. The percentages assigned to each criterion are depicted in Fig.1.

Therefore, it is the need of the hour to determine which admission criteria (MDCAT, MMI, FSC and Matric scores) better predicts the academic performance of students in medical college and be given higher weightage while calculating final aggregate. Our hypothesis is that the higher scores obtained in admission criteria are associated with better academic performance in medical college. The objective of the study was to determine the relationship between admission criteria examinations and performance of students in medical pre-clinical (1^st^ and 2^nd^ year MBBS) and clinical years (3^rd^, 4^th^ and 5^th^ year MBBS).

## METHODS

This correlational study was conducted on the data of students admitted in Shalamar Medical and Dental College (SMDC) in 2015 to determine the correlation between performance of students in examinations considered as admission criteria (scores obtained in SSC, HSSC, MDCAT and MMI) with their performance in medical college (scores obtained in pre-clinical and clinical years of MBBS). The examinations considered as admission criteria were taken as independent variables whereas the pre-clinical and clinical professional examinations were taken as dependent variables. The data from 2015 was taken because MMI was conducted this year and the students from this batch have already graduated. This means that the data of their professional exams (pre-clinical and clinical) was available for the study.

All the students who got admission in SMDC in the year 2015 were included in the study. The students who dropped out or transferred to another institute before graduating were excluded from the study. Students who had taken Scholastic aptitude test (SAT) instead of MDCAT were also excluded from the study. A total of 289 students were shortlisted for MMI in the year 2015. Every candidate had to take six short interviews with different interviewers. The pattern of the interviews was structured with case scenarios based on communication skills, teamwork, ethical decision making, critical thinking, empathy and motivation to study medicine constructs. Each interview lasted for six minutes. The total marks of the interview were 72 and the marks obtained by the student were then converted to out of four as it was decided that MMI was to be given 4% weightage.

### Ethical Approval

After receiving approval from the Institutional review board on 2/12/2021 (SMDC-IRB/AL/171/2021), the data was obtained from student’s affairs department with the permission of the principal. The data included scores obtained by each student in Matriculation or SSC (Secondary School Certificate SSC), FSC or equivalent (Higher Secondary School Certificate HSC), MDCAT (Medical and dental college admission test), MMI and five MBBS professional exams. All the marks were converted into percentages before analyzing and correlating the scores. The O and A level scores were used according to equivalence scores provided by HEC.

This study determined the correlation of MDCAT, MMI, FSC and Matric scores with mean scores obtained in the Annual professional exam (pre-clinical and clinical years).

1. MDCAT scores
2. FSc. (or equivalence scores)
3. Matric (or equivalence scores)
4. MMI scores

### With:

1. Mean scores of pre-clinical (1^st^ and 2^nd^ year) MBBS professional exam
2. Mean scores of clinical (3^rd^, 4^th^ and 5^th^ year) MBBS professional exam

The scores of each assessment were correlated with the result of all five professional exams (divided into pre-clinical and clinical years). The five year result included each student’s performance in MCQ, SEQ, OSPE/OSCE and viva as all of these assessment strategies reflect the overall academic performance of a student. IBM SPSS statistics 20 was used to analyze the data. Spearman’s correlation analysis was done because data did not follow a normal distribution. Multiple linear regressions were run to predict professional scores. Colinearity of independent variables in multiple models did not exceed 0.5 fulfilling the assumption of inclusion. P value of less than 0.05 was considered significant in this study.

## RESULTS

Data of 135 students was obtained but since 11 students had taken SAT exam instead of MDCAT so they were excluded from the study. Therefore, the data of a total of 124 students was included in the analysis. Table-I shows that there was a positive correlation between the independent variables (scores obtained in Matric, MDCAT, MMI and combination of all independent variables) and the dependent variables (scores obtained in pre-clinical and clinical years). The scores obtained by students in pre-clinical years correlated significantly with scores obtained by students in MDCAT (0.24, p = 0.007). It was also noted that there was a strong significant positive correlation (0.756) between scores obtained by students in pre-clinical years with scores obtained by students in clinical years (p = 000). Similarly, a significant positive correlation was observed between scores obtained in FSC and scores obtained in MDCAT (0.551, p = 000).

**Table-I:** Correlation (Spearman’s) between predictors and scores of students in clinical and pre-clinical years. (*Correlation is significant at 0.05).

Variables	Matric	FSC	MDCAT	MMI	Matric+FSC+MDCAT+MMI
Pre-clinical	0.04 (0.657)	-0.08 (0.384)	0.24* (0.007)	0.013 (0.884)	0.15 (0.096)
Clinical	0.113 (0.21)	-0.02 (0.823)	0.105 (0.244)	0.084 (0.354)	0.15 (0.095)

Spearman’s correlation analysis was done on different combinations of independent variables. It was observed that a combination of MDCAT and MMI scores correlated significantly (0.25, p = 0.006) with scores obtained by students in clinical years. Regression analysis was carried out to further explore the relationship between the independent and dependent variables. Multiple regressions analysis was done to determine the contribution of scores obtained in matric, FSC, MDCAT and MMI in predicting the performance of students in pre-clinical and clinical years (Table-II).

**Table-II:** Linear regression analysis of independent variables (admission criteria) and dependent variable (Scores obtained in pre-clinical and clinical exams).

Variables	Multiple Linear (pre-clinical years)			Multiple Linear (clinical years)		
	*B*	*Std error*	*P value*	*B*	*Std error*	*P value*
Matric	0.08	0.096	0.407	0.147	0.078	0.06
FSC	-0.25	0.15	0.099	-0.25	0.12	0.047*
MDCAT	0.177	0.083	0.035*	0.053	0.07	0.43
MMI	0.02	0.065	0.762	0.052	0.053	0.33

The analysis confirmed that MDCAT remained the most consistent predictor of students’ performance in pre-clinical years as it showed a positive significant relationship (coefficient = 0.177, p = 0.035). The relationship between performance in FSC and clinical years became significant in multiple regression models. There was a negative significant relationship between scores obtained in FSC and the students’ performance in clinical professional exams (coefficient = -0.25, p = 0.047).

## DISCUSSION

Admissions to Medical Colleges are a challenge for all stakeholders every year. Due to the high stakes and expense any attrition is costly. Traditionally prior academic scores were considered a sufficient criterion for admission. A lack of standardization among various academic programs and a drive for more focused filter for admitting the most suitable candidates, various Medical College admission Tests were devised (UKCAT, MCAT, MDCAT). Still concern was shown in the limited potential of such tests in predicting future performance. There was a need to identify admission criteria that determines the academic performance in medical college. It is interesting to note that in this study, the significant positive correlation MDCAT is restricted to the first two preclinical years and does not extend to clinical years.

The authors believe that since both, the MDCAT and preclinical professional exams assess cognitive aspects so a significant relationship was noted. Literature is divergent regarding the predictive validity of admission tests. This finding coincides with the findings of another study conducted locally where MDCAT showed positive correlation with academic performance of students in Part-1 FCPS examination since Part-1 exam also assesses cognitive aspects.^13^ Several studies show the reliability and predictive validity of Admission Tests.^14^,^15^ Our results get further weightage from the fact that, on multiple linear regression tests, MDCAT is again significantly correlated with preclinical years.

Another interesting finding in the present study is a significant positive correlation between the combined score of MDCAT and MMI and combined score achieved in clinical years. MDCAT and MMI scores alone did not correlate significantly with scores in clinical years. During the clinical years the non-cognitive attributes become increasingly applicable as students encounter actual patients along with the cognitive learning. Students possessing empathy, perspective, motivation, communication and social skills are more likely to excel.

These skills were assessed in SMDC’s MMI. This finding stands as a testimony to incorporating MMI’s in the selection process. It also validates the blueprint of MMI’s used during our admissions. A study in South Korea has demonstrated significant correlation between MMI total score and academic achievements based on performance based tests in the first two years.^16^ This could be due to the difference in curriculum as the institute in which this study was conducted had incorporated values, problem solving, professionalism, presentation and communication skills in the first two years; constructs that are aptly assessed by MMI. University of Dundee, UK also reported that MMI scores predict OSCE exam scores in the first two years at their medical school.^17^ The subjects taught and the OSCE examination conducted in Dundee are practiced in clinical years in our setup.

Another finding in our results was a significant negative correlation of FSc Scores with clinical year scores on multiple linear regressions only. Our finding does not compare with the previous study where FSc had a weak positive correlation with academic performance^12^. This difference might be due to the fact that the FSc scores are not standardized and various different examining boards are present in the country.

Medical students enrolled in SMDC are never from the same examining board. There are also students who study O-Levels and A-Levels and have to apply for an equivalence result to get their grade result converted to a score result. Therefore FSc scores as an independent variable have lots of variation in itself and unless a standardized format is used the results cannot be generalized. This was reiterated by a previous study as well which showed negative correlation of FSc scores with Part-1 FCPS exam scores. Again, the reason cited was the variability and lack of standardization in FSc exams.^13^

We also observed a significant correlation between the aggregate scores of the first two preclinical years and last three clinical years. This reflects a strong trend of academic achievement continuity during the course of five years. This is plausible because academic performance is affected by a complex interaction between both cognitive and non-cognitive factors.^18^ This awareness led to increasing acceptability and adoption of a more holistic admissions approach with expanded criteria in selection of students and an increasing recognition of importance of interviews during the high stakes Medical College admission process. Hence a number of health professions education programs started to use the multiple mini interview (MMI) to assess non-cognitive skills as part of the admissions process.^19^

### Limitations

This study was conducted using data of a single institute. Hence, the results cannot be largely generalized.

## CONCLUSION

Out of all the admission criteria employed to assess the merit of students, only MDCAT and MMI predict the academic performance of the students. FSC. negatively impacts the academic performance owing to lack of standardization. Therefore, our hypothesis is partially accepted as higher scores obtained in two admission criteria predict better performance of students in medical college. This study reiterates the belief that while selecting students for medical colleges the merit should be based on the assessment of cognitive and non-cognitive aspects. The cognitive assessment should be standardized and a uniform test should be taken by all candidates.

### Recommendations

Further research regarding impact of all these variables and their effect on MBBS academic performance should be undertaken with multiple college data to make more reliable claims. Secondly the relationship between each construct assessed by MMI and academic performance should be determined to figure out which constructs predict the performance better.

### Authors Contribution:

**SI:** Study conception and design, data acquisition and analysis, Manuscript writing.

**KMC:** Manuscript writing, editing and interpretation of data.

**IA:** Manuscript writing, editing and interpretation of data.

All the authors are responsible and accountable for the accuracy or integrity of the work.
